# Repeatability of radiographic assessments for feline hip dysplasia suggest consensus scores in radiology are more uncertain than commonly assumed

**DOI:** 10.1038/s41598-022-18364-9

**Published:** 2022-08-17

**Authors:** Elisabeth Ball, Margareta Uhlhorn, Per Eksell, Ulrika Olsson, Åsa Ohlsson, Matthew Low

**Affiliations:** 1 University Animal Hospital, Swedish University of Agricultural Sciences, Uppsala, Sweden; 2 XL Vet AB, Postvägen 7, Örbyhus, Sweden; 3 PawPeds, Gagnef, Sweden; 4 Department of Animal Breeding and Genetics, Swedish University of Agricultural Sciences, Uppsala, Sweden; 5 Department of Ecology, Swedish University of Agricultural Sciences, Uppsala, Sweden

**Keywords:** Diseases, Signs and symptoms, Diagnostic markers

## Abstract

Variation in the diagnostic interpretation of radiographs is a well-recognised problem in human and veterinary medicine. One common solution is to create a ‘consensus’ score based on a majority or unanimous decision from multiple observers. While consensus approaches are generally assumed to improve diagnostic repeatability, the extent to which consensus scores are themselves repeatable has rarely been examined. Here we use repeated assessments by three radiologists of 196 hip radiographs from 98 cats within a health-screening programme to examine intra-observer, inter-observer, majority-consensus and unanimous-consensus repeatability scores for feline hip dysplasia. In line with other studies, intra-observer and inter-observer repeatability was moderate (63–71%), and related to the reference assessment and time taken to reach a decision. Consensus scores did show reduced variation between assessments compared to individuals, but consensus repeatability was far from perfect. Only 75% of majority consensus scores were in agreement between assessments, and based on Bayesian multinomial modelling we estimate that unanimous consensus scores can have repeatabilities as low as 83%. These results clearly show that consensus scores in radiology can have large uncertainties, and that future studies in both human and veterinary medicine need to include consensus-uncertainty estimates if we are to properly interpret radiological diagnoses and the extent to which consensus scores improve diagnostic accuracy.

## Introduction

Discrepancies in radiological evaluations are common amongst radiologists^1,2^, with a wealth of studies in both human and veterinary medical fields documenting surprisingly large intra- and inter-observer assessment variation derived from the same radiographs (e.g.^1,3–12^. It is becoming increasingly apparent that this variation between and within observers occurs because of natural uncertainty in the process of interpreting radiographs, as well as ubiquitous factors that contribute to radiological error^1,2,13^. The degree of ‘error’ associated with radiological diagnoses often varies with the type of disease or diagnostic criteria used within screening programmes^2^. Thus, it is important to quantify the magnitude of this variation in a context-specific way and investigate how different processes may influence error generation. This is so uncertainty can be properly incorporated into clinical assessments, as well as identifying processes that may improve diagnostic certainty^2,13^. One of the most common methods used in disease-screening programmes to reduce the effect of inter-observer variation is the creation of a ‘consensus’ score based on the opinions of multiple observers^1,8,14^.

Consensus is generally interpreted as two or more radiologists agreeing on the interpretation or diagnostic score assigned to a radiograph^15^. This score is then often used as a standard of reference that represents the ‘true’ state of the condition in that individual as agreed upon by a consensus of experts^1,14,15^. While it appears reasonable to conclude that agreement amongst experts is a solid criterion for diagnostic accuracy, it is also important to understand what major assumption is being made when drawing such a conclusion. This assumption is that increased precision (i.e. agreement between observers) necessarily leads to improved diagnostic accuracy. However, reduced inter-observer variation could result from a situation where the experts are all equally wrong in their assessment^1,15^, and thus consensus does not improve diagnostic accuracy^11^. Although this may seem an unlikely scenario, the extent to which consensus grading is biased or shows variation from one assessment to another has not been rigorously assessed. Thus currently, consensus methods are being used to collate ‘error-prone’ individual data, but without knowing how much a consensus score is likely to differ if reassessed by the same or different radiologists. Consensus results are often considered a gold standard (e.g.^10,14^), but if they are less reliable than we assume this could create serious issues in our understanding of clinical assessment accuracy, the development of diagnostic criteria that minimise error rates, and the accuracy of artificial intelligence (AI) systems trained using these data^16^.

In veterinary medicine, hip dysplasia is a pervasive polygenetic disease where radiology is used to not only diagnose the condition, but to make recommendations regarding an animal’s breeding suitability^17–20^. Thus it is important to understand the limitations and uncertainties attached to radiological assessments in these animals; to date this has primarily been undertaken in dogs (e.g.^5,6,8,9^). Cats have a prevalence of hip dysplasia comparable to dogs (summarised in^20^) and share many of the radiographic findings common to canine hip dysplasia (with some species-specific differences)^21^. This suggests similar variation within and between radiological assessments for feline hip dysplasia as is seen for dogs. The management of feline hip dysplasia (FHD) is currently based on two international registries that assess radiographs for FHD: the Orthopedic Foundation for Animals (OFA) in North America and the Swedish-based PawPeds. These two organisations grade the degree of FHD according to the phenotypic radiographic appearance of the coxofemoral joint on a standard extended ventrodorsal projection. Both programmes use an ordinal grading system relating to joint conformation and secondary degenerative changes^14,18,20^. Radiographs submitted to OFA are independently evaluated by three board-certified radiologists out of a pool of consultants and a consensus grade (based on a median of these three evaluations for both hips combined) is the official FHD score in cats above 24 months of age^14,18^. This consensus score is viewed as a ‘gold standard’ accurate assessment^14^. In the PawPeds screening program, a single veterinary radiologist employed by the programme evaluates and separately grades each hip from radiographs of cats above 10 months of age.

This study focuses on data from the PawPeds health programme and the subsequent FHD assessments of three veterinary radiologists in order to answer questions regarding the reliability of FHD assessments in cats, the factors that might influence this reliability, and whether consensus scores are as accurate (or repeatable) as is currently assumed. For this, sampling was restricted to the Maine Coon, as this is the breed with the most cats screened in health programmes^20^. Based on repeated individual FHD assessments and a mutual consensus grade by the three radiologists, we asked the following questions: (1) what is the intra- and inter-observer variation in the assessment of FHD radiographs? (2) to what extent is this variation related to the cat’s age (since OFA assesses cats > 24 months while PawPeds assesses cats > 10 months) or radiographic quality? (3) what is the probability of a cat’s FHD score changing in any particular direction in a reassessment, given its initial hip score? (4) what is the probability of a consensus score changing between the first and second assessment; and how does the definition of consensus influence this repeatability (e.g. independent majority vs. independent unanimous vs. mutual consensus via discussion)? and (5) how does the initial consensus score influence the predicted direction and magnitude of any potential change in the consensus grade upon re-evaluation? These questions are not only of interest for researchers working with FHD or users of the PawPeds programme, but highlight general issues about our assumptions relating to how we interpret the accuracy of individual and consensus scores in diagnostic radiology.

## Results

### Within- and between-observer repeatability

Intra-observer agreement in FHD scores between the first and second assessment showed some minor variation between observers, with approximately 65–71% of scores in the first assessment being the same as individual scores given during the second assessment (Tables 1 and 2). Changes in FHD scores between the first and second assessments were more likely to occur between scores 1 & 2 (30% of all changes were from 1 to 2 and 18% of all changes from 2 to 1) and scores 0 & 1 (28% of FHD score changes from 0 to 1, and 7% of changes from 1 to 0; Table 1). Inter-observer agreement was slightly lower than the intra-observer agreement during the first assessment round (~ 63%); however agreement between observers was higher during the second assessment round (~ 77%; Table 2).

**Table 1:** Hip evaluations for 196 hips from 98 cats as scored by three independent veterinary radiologists (Observers 1–3). Each hip was given a score based on four categories (Hip Score (HS) 0–3) and the same observers scored all hips on two separate occasions (i.e. First assessment and Second assessment). In the upper half of the table, data are presented as both the absolute counts of hips within each hip score category (Abs) and as a frequency relative to the total number scored by each observer in each category during each occasion (Freq = Abs/196). In the lower half of the table we present the intra-observer changes in their hip scores between the first and second assessments. These data include how hip scores changed from their initial value, their magnitude and direction (e.g. a change from a score of 0 to 1 from the first to second occasion is HS 0 to 1). Here these intra-observer changes are also presented as the absolute numbers of changes in hip score, and the frequency of these changes (Freq) relative to the total number of hips that changed score for each individual observer (Observer 1 N = 72; Observer 2 N = 56; Observer 3 N = 55).

	Observer 1		Observer 2		Observer 3	
	Abs	Freq	Abs	Freq	Abs	Freq
**First assessment**						
HS 0	66	0.336	53	0.270	73	0.372
HS 1	83	0.423	65	0.332	64	0.326
HS 2	38	0.194	59	0.301	44	0.224
HS 3	9	0.046	19	0.097	15	0.076
**Second assessment**						
HS 0	46	0.235	46	0.235	59	0.301
HS 1	86	0.439	76	0.388	67	0.342
HS 2	43	0.219	52	0.295	49	0.250
HS 3	21	0.107	22	0.112	21	0.107
**Increased hip score**						
HS 0 to 1	22	0.305	12	0.214	17	0.309
HS 1 to 2	25	0.347	12	0.214	17	0.309
HS 2 to 3	12	0.167	4	0.071	7	0.127
HS 0 to 2	1	0.014	2	0.036	2	0.036
HS 1 to 3	0		0		0	
HS 0 to 3	0		0		0	
**Decreased hip score**						
HS 1 to 0	3	0.042	7	0.125	3	0.054
HS 2 to 1	9	0.125	18	0.321	6	0.109
HS 3 to 2	0		1	0.018	1	0.018
HS 2 to 0	0		0		2	0.036
HS 3 to 1	0		0		0	
HS 3 to 0	0		0		0

**Table 2:** Intra- and inter-observer agreement probabilities for the three observers (Obs 1–3) across 196 hip assessments. The intra-observer agreement refers to the probability of an individual observer recording the same FHD hip score during the first and the second assessments. The inter-observer agreement probabilities refer to paired observer comparisons (e.g. Obs 1 -2 is the agreement between observer 1 and observer 2) during the first assessment (1st) and the second assessment (2nd). We report the mean and its standard deviation, the median and the 95% CI as estimated from a binomial GLMM to estimate the probability of between-score agreement.

Agreement	Mean	SD	Median	95% CI
**Intra-observer**				
Obs 1	0.648	0.035	0.649	0.57–0.71
Obs 2	0.707	0.029	0.706	0.65–0.76
Obs 3	0.712	0.029	0.711	0.66–0.77
**Inter-observer 1st**				
Obs 1–2	0.648	0.029	0.648	0.59–0.71
Obs 1–3	0.629	0.029	0.630	0.56–0.68
Obs 2–3	0.635	0.028	0.636	0.58–0.69
**Inter-observer 2nd**				
Obs 1–2	0.778	0.025	0.777	0.73–0.83
Obs 1–3	0.770	0.025	0.771	0.72–0.81
Obs 2–3	0.759	0.026	0.761	0.70–0.81

### Individual repeatability conditional on the original hip score

**Figure 1:**
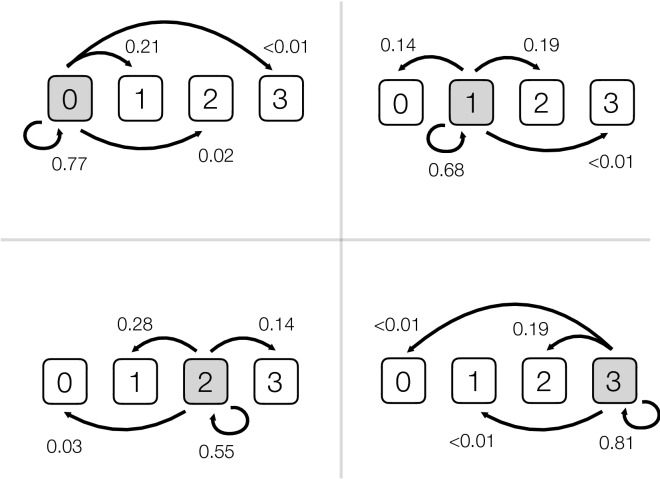
Conditional probability estimates for a ‘re-evaluation’ of an FHD score from an individual radiologist, given that the same radiograph is assessed without reference to the original score by either the same person or a new person. In each panel the grey box indicates the original hip score grade (top left original score = 0, top right = 1, lower left = 2, lower right = 3), and the arrows and associated numbers show the estimated conditional probabilities of hip scores from a re-evaluation. For example, in the top right panel an original hip score grade = 1 was given (shaded grey). The multinomial model predicts that during a reassessment there is a 68% chance of the same score being given, a 14% probability that the hip score changes to 0, a 19% probability that the hip score changes to 2, and less than 1% that the hip score changes to 3. These probabilities are based on combined data from the intra- and inter-observer repeated observations and are conditioned on data that satisfy the original score (i.e. Pr (new observation | original hip score); see Table 3 for the uncertainties attached to these estimates).

The multinomial analyses showed that the probability of a hip being given a specific score (0, 1, 2 or 3) during a repeat assessment by an individual radiologist was conditional on the original score assigned to that hip (Fig. 1; Table 3). Hips that were originally scored at the lower and upper end of the hip-score range (i.e. 0 or 3) had higher repeatability (0.77 and 0.81 respectively) than hips in the middle of the range (hip scores 1 & 2; repeatability of 0.68 and 0.55 respectively). For hips that changed score during reassessment, nearly all of them changed by only a single value; with the direction of this change contingent on the original score (i.e. scores of 0 could only increase, scores of 3 could only decrease, while scores of 1 and 2 were split in both directions; Fig. 1; Tables 1 and 3). Interestingly, the only change that occurred with a > 1 unit change in FHD score occurred between categories 0 and 2; there was a 2–3% probability of a hip changing its score between these two categories during a reassessment (Fig. 1; Table 3).

**Table 3:** Probabilities of getting an FHD score in a second assessment, conditional on the FHD score given in the first assessment (Original = 0, 1, 2 or 3) for: (a) an individual radiologist, (b) a majority consensus score (i.e. at least 2 out of 3 radiologists agree on the score in both assessments), and (c) a full consensus score (i.e. 3 / 3 radiologists agree on the score in both assessments). Probabilities (represented as a proportion between 0 and 1) for all possible outcomes are presented as the mean and 95% Bayesian CIs as generated from multinomial modelling of the data collected in this study. The left column shows the FHD score in the first and second assessments: e.g. if a hip was scored 0 in the first assessment, and 2 in the second assessment, this is represented as 0 to 2. The probability of agreement between the first and second assessment is highlighted in grey for each possibility. For a visual representation of the individual and full consensus results see Figs. 1 and 3, and for the majority consensus see Fig. S1. For the estimates associated with ‘median’ consensus repeatability see Table S2.

FHD score	Individual		Majority consensus		Full consensus	
	Mean	95% CI	Mean	95% CI	Mean	95% CI
**Original = 0**						
0 to 0	0.765	0.73–0.79	0.729	0.61–0.83	0.883	0.77–0.96
0 to 1	0.211	0.18–0.24	0.266	0.16–0.38	0.116	0.04–0.22
0 to 2	0.023	0.01–0.03	0.001	0–0.016	0.000	0–0.001
0 to 3	0.001	0–0.004	0.001	0–0.015	0.000	0–0.001
**Original = 1**						
1 to 0	0.135	0.11–0.16	0.044	0.01–0.10	0.003	0–0.034
1 to 1	0.675	0.64–0.70	0.728	0.62–0.83	0.825	0.67–0.94
1 to 2	0.189	0.16–0.21	0.226	0.13–0.32	0.171	0.06–0.32
1 to 3	0.001	0–0.003	0.001	0–0.01	0.000	0–0.001
**Original = 2**						
2 to 0	0.032	0.02–0.06	0.002	0–0.02	0.001	0–0.002
2 to 1	0.283	0.24–0.33	0.214	0.10–0.35	0.064	0.002–0.21
2 to 2	0.548	0.49–0.59	0.685	0.54–0.81	0.869	0.67–0.98
2 to 3	0.136	0.10–0.17	0.097	0.03–0.20	0.065	0.002–0.21
**Original = 3**						
3 to 0	0.003	0–0.013	0.005	0–0.05	0.001	0–0.004
3 to 1	0.003	0–0.014	0.006	0–0.06	0.001	0–0.005
3 to 2	0.187	0.14–0.24	0.012	0–0.08	0.017	0–0.169
3 to 3	0.806	0.75–0.85	0.976	0.87–0.99	0.979	0.81–1.0

### External factors related to repeatability of individual assessments

**Figure 2:**
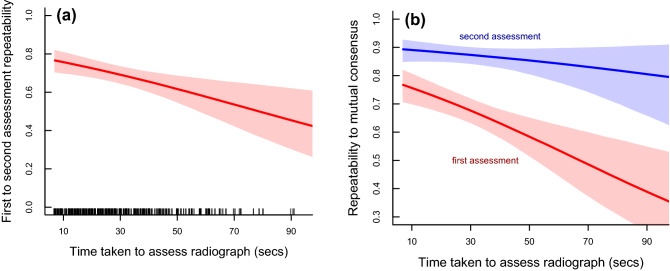
Relationship between repeatability of radiographic scoring of feline hip dysplasia and the time taken (in seconds) to assess the radiograph; (a) shows this pattern averaged for the three evaluators, for repeatability between the first and second assessment; (b) shows the pattern for repeatability between the individual scores of the first assessment and the mutual consensus score (red) and from the second individual assessment and the mutual consensus score (red). Lines represent mean predictions from Bayesian GLMMs and shaded areas the 95% CIs of the predictions. In panel (a) the times for all hip evaluations are shown as a rug plot along the x-axis.

There was little evidence of the cat’s age or the quality of the radiograph playing a role in the repeatability of radiographic scoring (Supplementary Table S1a–c). However, there was a clear relationship between the time taken by each evaluator in assessing a radiograph in the first assessment and the repeatability of that score in their second assessment (Fig. 2a). Here as more time was taken to assess the radiograph, the repeatability declined; suggesting that time taken was an indicator of uncertainty in the evaluation. This relationship was also seen when comparing agreement of the evaluators' scores during the first assessment to the final mutual consensus score, but not for comparing the second assessment to the mutual consensus scores (Fig. 2b).

### Consensus

During the first reading, independent unanimous consensus (i.e. all 3 observers agreed) was reached in 93/196 (47%) hips evaluated, while during the second reading this consensus occurred in 128 hips (65%). Almost 30% of the hips (27/93) that achieved unanimous consensus during the first reading, failed to reach this level of consensus in the second reading (this number is almost 50% (62/128) if the comparison is made in the reverse direction; Appendix S2). This resulted in only 66 hips achieving independent unanimous consensus in both readings. Interestingly, 6 of these 66 hips (9%) changed their unanimous consensus score between readings (Appendix S2). Differences between the first and second independent consensus readings were also evident when comparing them to the final mutual consensus score reached by discussion (which was agreed upon immediately after the second assessment). Here we found that while all second-reading unanimous consensus scores (128/128) agreed with the final mutual consensus score, only 77 of the 93 first-reading unanimous consensus scores (83%) agreed with the final mutual consensus score. Of the 16 that did not agree, the majority changed from 0 to 1 (6 cases) or from 1 to 2 (6 cases). The other four changes were from 2 to 3 (2 cases), 0 to 2 or 2 to 1 (1 case each; Appendix S2). This difference between the first and second readings in relation to the final consensus score was also evident when comparing each evaluator’s scores to the mutual consensus score; the level of agreement to the mutual consensus score was much lower for all evaluators during the first reading, than compared to the second (Appendix S2).

If the definition of consensus was relaxed to a minimum of 2 observers agreeing on a score (i.e. a majority decision), then independent consensus was reached in 189 / 196 hips (96%) during the first assessment and in all 196 hips during the second assessment. The agreement in these majority consensus scores between the first and second assessments was 75%, meaning that one quarter of all majority consensus scores changed their score between the first and second readings (36 consensus scores increased by 1, and 12 decreased by 1; Appendix S2). If these independent consensus scores are compared to the final mutual consensus score, then agreement between majority independent and mutual consensus scores were 71% and 94% for the first and second assessments respectively (Appendix S2).

### Repeatability of consensus scores conditional on the original consensus score

**Figure 3:**
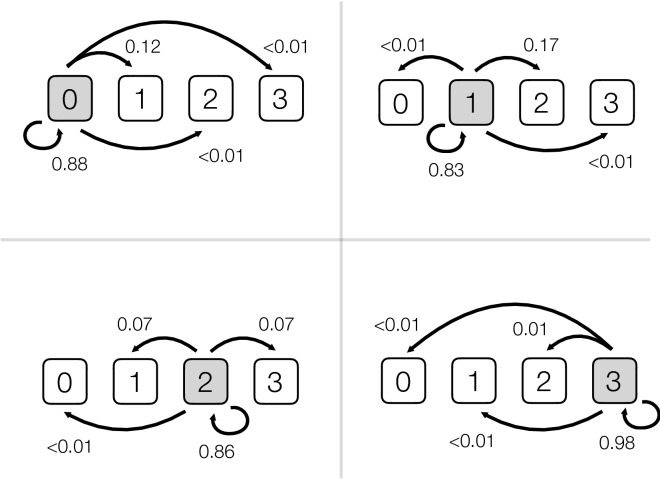
Conditional probability estimates for the full independent consensus FHD score from the first assessment (i.e. all observers independently agreed on the score; N = 93) corresponding to the mutual consensus FHD score after the second assessment. In each panel the grey box indicates the original independent consensus hip score grade during the first assessment (top left original FHD score = 0, top right = 1, lower left = 2, lower right = 3), and the arrows and associated numbers show the estimated probabilities of the mutual consensus hip scores after the second assessment from the multinomial model. For example, in the top right panel the original full consensus score = 1. Our modelling predicts that on reassessment there was an 83% chance of the observers reaching the same consensus score, a 17% probability that the consensus score would change to 2, and < 1% probability of consensus changing to 0 or 3. These probabilities are based on the full consensus data collected in the first assessment and comparing these to the mutual consensus scores reached in the second assessment: i.e. Pr (second consensus | first consensus). See Table 3 for the uncertainties attached to these estimates.

The probability of an independent consensus score during the first assessment being given the same independent consensus score upon reassessment was conditional on the definition of consensus (i.e. median value of the 3 observers, majority (2/3 observers agree) or full consensus (3/3 agree)), in addition to the original consensus score (Tables 3 & S2; Appendix S2). Hips with a consensus score of 3 were highly repeatable on reassessment (~ 98% probability of reaching the same consensus score) regardless of how consensus was defined (Tables 3 & S2; Appendix S2). However consensus grades of 0 to 2 were more prone to disagreement during the repeated assessment (consensus score 0 agreement range = 0.729–0.883; score 1 = 0.679–0.825; score 2 = 0.685–0.869). Here the highest levels of agreement were if an independent unanimous consensus was reached in the first assessment; but note that even under these restricted conditions of all observers independently agreeing when first grading the radiographs, there was still a 12–17% probability of the consensus score changing on the second assessment (Table 3; Fig. 3; Appendix S2).

## Discussion

In this study we show that intra- and inter-observer agreement on diagnostic scoring of FHD radiographs suffers from the same issues of repeatability as has been found not only in canine hip dysplasia^4,5,8,9^, but more generally with radiographic disease interpretation in animals and humans^7,12,13,22^. To place this observational uncertainty into a practical context, we also quantified the direction and magnitude of what could be expected if a ‘second opinion’ on a hip assessment was sought within this programme, and the degree to which consensus scoring improves diagnostic repeatability. We show that any predicted changes in a repeated assessment are not random, but rather were dependent on the original hip score grade given. As has been found previously, scores at the extreme of the diagnostic range (i.e. 0 or 3) were more likely to be scored the same on reassessment when compared to those in the middle (i.e. FHD score = 1 or 2; see also^10^). This likely reflects the greater certainty associated with interpreting the diagnostic criteria when extremes are viewed (e.g. no pathology and maximum pathology), as well as a simple statistical fact that scores at the ends of a range can only change in one direction, while those in the middle of the range can change in two directions. An understanding of why radiographic repeatability is not perfect and how these patterns of uncertainty should be interpreted in the context of an assessment is an important aspect of the diagnostic process: not only for practitioners, but also for clients and disease-screening programmes that use these scores to make decisions regarding the use of specific animals for breeding^2,13^.

We caution against a naive interpretation of imperfect repeatability in FHD assessments. For some people this may be seen as a signal that they can selectively choose the diagnosis that suits them or bypass disease-screening programmes altogether because the assessments are not perfect. For others, our results may be seen as a need to find assessment criteria that are more ‘definitive’ for the disease and lead to minimal disagreement between observers. But this misunderstands the nature of uncertainty and accuracy when making decisions, especially the interpretation of radiographs^2,13^. In the case of FHD scoring, the aim is to assess the probability that a cat is carrying genes for a disease, and to subsequently limit its breeding potential based on this probability^14,20^. To do this we use a two-dimensional x-ray image that represents a three-dimensional physical object, which itself is constructed from a complex interplay of an animal's genome, the environment it was exposed to during its development *and the hip dysplasia genes we are interested in*. Thus the diagnostic approach from the very beginning needs to acknowledge this uncertainty, in that a radiograph is an abstraction that only provides information on the probability of an animal carrying FHD genes (e.g. based on heritability estimates related to phenotypes^20^). In the same way, a radiologist’s assessment of that radiograph includes an additional layer of interpretation in translating whether a particular animal carries FHD genes. This means there is no definitive answer to a question like, "If my cat has an original FHD assessment score of 2 and a second opinion gives it a score of 1, does that mean it is ok to breed from that cat?” Like a weather forecast predicting rain, these assessments should be seen as a probability of a cat carrying FHD genes. We accept that weather forecasts are not perfect, yet we can still use their predictions to make informed choices. It is no different with radiological diagnoses. For example, if a cat receives three different FHD scores (e.g. 0, 1, 2) this simply means there is a lot of uncertainty in deciding whether it carries FHD genes or not. In such a situation the client should not take this to mean it is fine to breed from the cat (because no-one can agree), but rather they should think carefully about the risk of them breeding from a cat where the FHD status is highly uncertain.

One approach to limiting the uncertainty introduced by radiological interpretation, is to use a consensus approach where multiple expert readers score a radiograph and a consensus is reached by either taking the median score, the majority score or a mutual agreement score decided after discussion^15,23^. Consensus approaches are seen as producing a high-quality reference standard^15,16^, the reference from which diagnostic ‘error’ is determined^2^, or providing an assurance to the correctness of the diagnosis^10,14^. While we agree that the opinions of multiple experts will tend to provide more information about the accuracy of a diagnosis than the opinion of one expert, our results show that consensus scores do not necessarily uncover the ‘true’ diagnosis from which error can be determined. There are three things to consider here. First, if consensus provides an assurance of accuracy in the diagnosis, then we should see almost perfect repeatability of consensus scores if they are retested. But as we show in this study, consensus scores can change from one assessment to another up to 30% of the time depending on how consensus is determined (majority versus unanimous agreement, and conditional on the initial grading; Table 3). Even when we restrict our consideration to unanimous consensus (i.e. all observers independently agree during an assessment), the probability of the same consensus score being reached on two separate occasions can be as low as 82.5%. Second, as we discuss in the previous paragraph, we should not only be seeking the best estimate of the diagnosis (by taking a median or mean), but we also need to include the variation in the expert opinions in order to understand how confident we can be in that consensus score^15,23^. Being told that your cat has a consensus hip score of 1 provides some information, but without knowing if the experts all agreed or if this is a median from a range of scores between 0 and 2 will significantly change your interpretation and confidence in that consensus score. Finally, we need to acknowledge that because the process that produces the image for assessment is uncertain (i.e. not all cats with FHD genes will show pathology, and radiographic image quality may mask pathology or contain artefacts that resemble pathology), radiologists may agree on consensus but without this relating to accuracy in the diagnosis^1,11,15^. Thus, while much emphasis has been placed on understanding uncertainty in individual radiological assessments^1,2,13^, the same level of understanding has not yet been extended to the limitations and uncertainties associated with consensus assessments^15^.

An example of how a deeper understanding of uncertainty and inaccuracy in consensus scores may dramatically change the interpretation of results, can be seen by comparing our study to Pulkinnen et al.^12^. In both studies the probability of achieving a full independent consensus score (i.e. all three radiologists agree) during an assessment was similar (56 versus 61%); as was a restricted consensus score where only two out of three grades needed to be identical during an assessment (98 versus 99%). In Pulkinnen et al.^12^, this near perfect probability of reaching a majority agreement during an assessment was then used to justify their radiological assessment criteria. However, this requires a strong assumption that consensus scores reflect diagnostic accuracy. In our study we did not assume this, and instead quantified how often these consensus scores changed between assessments; here repeatability was as low as 68% depending on the original majority consensus grade (Table 3). But what causes this low consensus repeatability despite an almost perfect probability of reaching a majority consensus? Imagine a radiograph where the pathology is a borderline FHD score between 0 and 1. The first radiologist decides to score it a 0, while the second radiologist scores it a 1. Regardless of how the third radiologist scores the radiograph (0 or 1), there is a guaranteed majority decision reached, independent of the ‘true’ diagnosis. Thus a near-perfect probability of achieving a majority decision during an assessment, does not say anything about the accuracy of the consensus score if one can get the same result by flipping a coin. This clearly shows that focussing on how often we can achieve consensus tells only half the story; it is crucial to also understand whether consensus is repeatable and how best to incorporate measures of uncertainty when describing consensus outcomes^15,23^.

We also examined relationships between assessment scores and a number of factors that could potentially influence their repeatability. There were two factors that the PawPeds programme has influence over, i.e. age of the cat and quality of the submitted radiograph; however neither of these demonstrated obvious effects on intra-observer repeatability. This suggests that despite there being a range of radiographic qualities submitted by veterinary practitioners, the current quality control process at PawPeds (where some submissions are rejected as ‘unreadable’) appears sufficient in excluding radiographs of such low quality that they interfere with FHD scoring. It is also worth noting that PawPeds allows cats as young as 10 months of age to be assessed, while the other major international FHD screening programme (OFA) assesses cats > 24 months of age^14^. We could not see any effect of age on radiographic assessment repeatability (i.e. young cats and older cats in our assessments had the same repeatability measures). Thus, from this perspective there is no evidence for PawPeds to change their minimum age requirements.

There were two additional factors that did have relatively large effects on the repeatability of FHD scores. The time taken for a radiologist to interpret a radiograph on the first assessment was negatively correlated to the intra-observer repeatability and to the mutual consensus score. We interpret this as not being related to the general quality of the radiograph, but to the fact that a 4-level category is being imposed on top of a series of continuous anatomical features (i.e. hip congruency, acetabular-femoral head overlap, and osteophyte formation). Thus when a hip is close to one of these category boundaries, it will take more time for the radiologist to decide in which category the hip belongs, and in turn be more likely to be placed in the adjacent category during a reassessment. This is supported by the second influential factor we examined, being the score assigned to the radiograph in the initial assessment. By looking at the probability of an FHD score being given at a second assessment, conditional on the first FHD score, we show there is much greater uncertainty in scores 1 and 2 compared to 0 and 3. Also, that changes in scores were almost always from one category to the adjacent one, as is suggested given our interpretation of the ‘time taken’ analysis above. This effect is not only for individual intra- and inter-observer repeatability, but also for the consensus scores where we see high repeatabilities for score 3 (and somewhat high for 0). As discussed earlier, this is important information in helping us understand how to interpret our confidence in an individual radiological assessment.

Our results suggest that in both human and veterinary radiology, greater attention now needs to be focussed on the uncertainties and repeatability associated with consensus scores and how to interpret these in the context of diagnostic assessments^2,15,23^. In this study we were able to calculate consensus uncertainty among the same observers using the same radiographs for assessment. However, this ‘intra-observer consensus’ is likely to have a higher repeatability than if consensus is compared between different groups of experts (an ‘inter-observer consensus’), as is seen for individual repeatability comparisons^1^. Also, calculating the accuracy of consensus approaches should consider using different radiographs from the same animals, to ensure that consensus is related to the pathology on display rather than specific aspects of the radiograph. Also, it is possible that repeatability effects may differ between breeds, and this should also be taken into consideration when assessing the value of consensus agreement. As was seen when comparing independent consensus within the first and second assessments to the final mutual consensus, there were dramatic differences in the level of agreement and the effect of time taken to read the radiograph. This suggests that the design of repeatability experiments and the time between independent assessments and mutual consensus assessments may have a large effect on estimates of uncertainty and repeatability of consensus scores. Thus care will need to be taken in future studies to ensure we get accurate estimates of the uncertainties surrounding consensus scoring. These estimates are critical if we are to fully understand what our radiological diagnoses mean, and the extent to which consensus scores improve the accuracy of diagnoses. This is becoming increasingly important as AI systems are being trained on radiographic data using consensus scores as their diagnostic reference^16^.

## Materials and methods

### The PawPeds feline hip dysplasia (FHD) screening programme

An international feline health programme to limit FHD was started in 2000 and is currently administered by PawPeds (https://pawpeds.com/healthprogrammes/). The programme grades the degree of FHD in individual cats > 10 months old according to the radiographic appearance of their coxofemoral joints on standard extended ventrodorsal projection radiographs. One veterinary radiologist is responsible for all evaluations within the programme and scores each hip separately according to four-grade scale: grade 0 = normal hip with no evidence of FHD; grade 1 = congruent hip with acetabulum covering < 50% of the femoral head or incongruent hip with acetabulum covering > 50% of the femoral head without secondary degenerative changes; grade 2 = mild osteophyte formation and/or incongruent joint in combination with < 50% of the femoral head covered by the acetabulum; grade 3 = moderate-severe osteophyte formation (see^20^ for radiographic examples of each). The programme bases its recommendations to breeders to limit FHD by selectively breeding from cats according to these grades. Cats with grade 2 or 3 on either hip should be excluded from breeding. Cats with grade 1 on either or both hips are recommended to only be mated to cats that have normal hip status (grade 0) in both joints. The recommendation by PawPeds to the veterinary radiologist is that if a hip score is borderline, it should be graded at the lower score: i.e. scores should be conservative in these cases. The PawPeds programme has been demonstrated as successful in reducing FHD expression in subsequent generations^20^.

### Experimental design

Radiographs from both hips of 98 Maine Coon cats (35 males and 63 females; a sex ratio similar to the PawPeds database;^20^) were retrieved from PawPed’s hip dysplasia screening programme database by an independent person who was not involved in the reading or analytical process. All cases had at least one extended ventrodorsal projection of the pelvis and coxofemoral joints specifically taken for FHD assessment. The age of all cats was known at the time of radiographic examination (mean = 18 months; range = 10–46 months) and had previously been graded within the PawPeds’ programme by a veterinary radiologist (PE) within the last 5 years. Cats were randomly selected from each FHD graded categories of archived results within PawPeds. The number of cats per category was chosen to ensure sufficiently large sample sizes to allow accurate estimation of agreement scores, probabilities of consensus conditional on the original FHD score, and to reflect the relative frequency of these hips scores in practice. The cases were organised alphabetically based on the cat’s registered name and were assigned a unique number that was linked to a separate folder containing the hip radiographs of each cat. Almost all radiographs were in DICOM format (using image viewing systems; Horos and ImageJ) with a few exceptions in JPEG.

The radiographs for each case were then evaluated and both hips for each cat were scored independently by three veterinarians; one first year veterinary radiology resident (observer 1), one board-certified veterinary radiologist with over 20 years of experience (observer 2) and one associate professor in veterinary radiology with over 20 years of experience (observer 3). All observers were familiar with the current hip reading protocol for PawPeds (two of the observers were either previously or currently employed by PawPeds to assess FHD radiographs for the programme (observers 1 & 3), and all three radiologists discussed the PawPeds grading criteria before this study). When reading the radiographs the observers were unaware of the patient’s history, age, sex and previous FHD grade. Each hip was scored by the veterinary radiologists on three separate occasions. For the first scoring the observers read the images in the same manner as would occur during a normal PawPeds screening procedure. For this the 98 cases (196 hips) were independently reviewed by the radiologists in 4 separate periods with 24–25 cases per session and approximately 14 days between each session to simulate the normal screening conditions within the program. Each coxofemoral joint was graded using the current PawPeds 4-level grading scale. During this reading the radiologists also recorded any issues they thought might impact on the reading of the radiograph (e.g. contrast or position of the animal^24^) and the time taken to make a decision on a score for that cat. A second assessment of the same 98 cases took place approximately 8 weeks after the first assessment was completed. At the second assessment, all three observers individually graded both coxofemoral joints of each cat on the same day and directly after each case they compared scores and decided on a consensus grade by mutual discussion (third assessment). The order of cases remained the same in all assessments. Observers did not report or discuss their results with the other observers during or between readings and they had no access to their first assessment scores at the time of the second assessment and mutual consensus decision.

### Statistical analysis

In addition to summary statistics, we used two main approaches for analysing repeatability in the hip scores assigned to each individual animal. The first was using a binomial GLMM framework to estimate inter-observer repeatability, intra-observer repeatability, and how scores related to the final consensus score; with these comparisons recoded to 1/0 data (i.e. did the hip scores agree or disagree). For the inter-observer analysis, each hip score (0–3) from one observer was compared to the scores given by each of the other two observers during each of the two sessions. For the intra-observer analysis, the scores of each observer from the first session was compared to their own score in the second session. These models were constructed so that the identity of each observer was included as a hierarchical level on the model’s intercept to allow repeatability estimates to be generated not only for the mean of all observers, but also separately estimated for each of the three observers (see Appendix S1 for a full model and data description). This allowed us to extend this basic model structure by adding explanatory variables within the GLMM framework to help our understanding of the possible causes of variation in repeatability of the observations. These included the time taken to read the radiograph in deciding a hip score, the age of the cat, and the ‘quality’ of the radiograph: i.e. if comments were made about problems with the radiographic quality or positioning of the cat.

We also evaluated the consistency of consensus scores from several perspectives in this study; we refer to these as ‘independent consensus’ and ‘mutual consensus’. For independent consensus we examined whether all three independent evaluations for a particular hip were in agreement during a reading when there was no communication between the observers. How often did independent consensus occur, and how stable was this consensus over time? Thus we examined how often independent consensus remained between the two independent readings, and also if any of these consensus scores changed between the first and second readings. Because consensus can also be defined as a ‘majority decision’ in clinical assessments^11,25^, we reran these analyses based on independent consensus being defined by the majority decision (i.e. at least 2 observers agree on the score, and this is considered the consensus score). This allowed us to estimate repeatability for unanimous (3/3) versus majority (2/3) definitions of consensus. For mutual consensus, we used the final consensus score that was reached for each hip via discussion between the three evaluators immediately after the second reading. This allowed us to not only compare individual evaluator’s scores during both readings to the mutual consensus score, but also compare the different ways of assessing consensus, and whether the ‘independent’ consensus changed in relation to the ‘mutual’ consensus as the study progressed.

The second analytical approach involved a 4-level multinomial modelling framework to estimate the specific probabilities of a particular hip score being given by a new observer (or a repeated assessment), given that a specific hip score was assigned in the original observation. This was to answer the following questions: (1) ‘what is the probability of a hip being given an FHD score of 0, 1, 2 or 3 in an individual radiologist re-evaluation, given its original hip score?’ and (2) ‘how repeatable is a consensus score (both full and partial) during a re-evaluation, given the original consensus score?’ Here we conditioned the data to the original observation or consensus score in the first evaluation. For each question we created four datasets based on subsets of individual or consensus hip scores; each of these was conditional on the four possible initial hip scores (i.e. individual or consensus score = 0, 1, 2 or 3). For the first question related to an individual reassessment, the count data for the multinomial categories included the subsequent score by that same observer during the repeated measurement, and the comparable scores by the other observers during the same observation period (we viewed this as if an owner were to ask the same radiologist to check their original assessment, or for a new radiologist to be consulted as a ‘second opinion’). For the second question related to consensus scores, the multinomial category data were the consensus scores in the second assessment. We ran multinomial models using each of these datasets, with these models estimating the probability of the four possible hip score categories being subsequently given (either by an individual observer or by a consensus assessment), conditional on the initial hip score (see Appendix S1 for a full model and data description). All analyses were undertaken in a Bayesian framework to allow us to extract the probabilities of interest. These were implemented in R^26^ using JAGS^27^ using minimally informative priors. All models were checked for convergence by visual inspection of stability and mixing of the chains and fit, using posterior predictive checks.

### Ethical statement

This study was conducted using pre-existing radiographs sourced from a voluntary health screening programme (PawPeds). Thus, no animals were involved in this study.

## Supplementary Information

Supplementary Information 1.

Supplementary Information 2.

Supplementary Information 3.

## Data Availability

The radiographs used in this study are available from PawPeds (https://pawpeds.com/healthprogrammes/). The raw data from the radiologists categorising the 196 hip scores across the three assessments can be found in the online supplementary material (Appendix S2).
