# Supplementary material for: Repeatability of radiographic assessments for feline hip dysplasia suggest consensus scores in radiology are more uncertain than commonly assumed
Source: Sci Rep. 2022 Aug 17;12:13916. doi: 10.1038/s41598-022-18364-9 (PMC9385612; doi:10.1038/s41598-022-18364-9)
Supplement: Supplementary file 2 — Supplementary Information 2. [file 41598_2022_18364_MOESM2_ESM.pdf]

Supplementary Appendix S1 for the manuscript:

# Repeatability of radiographic assessments for feline hip dysplasia suggest consensus scores in radiology are more uncertain than commonly assumed

By Elisabeth Ball, Margareta Uhlhorn, Per Eksell, Ulrika Olsson, Åsa Ohlsson & Matthew Low

In this file is the following information:

Appendix 1a: Binomial model formulation for intra- and inter-observer repeatability
Appendix 1b: Binomial model formulation including explanatory variables

Appendix 1c: Multinomial model formulation for calculating FHD score probabilities conditional on the initial hip score given

**Appendix 1a:** Binomial model formulation for intra- and inter-observer repeatability

Here we used a 1/0 response variable (‘agree’) based on either the repeated assessment by the same observer (intra-observer analysis) or by paired agreements between the three observers to estimate the inter-observer repeatability (p). The subscript ‘i’ refers to the individual data comparisons for each hip, and the subscript ‘j’ refers to the grouping level (j = 3) around which the hierarchical nature of the model is constructed (and subsequently estimated). These ‘j’ grouping levels for the intra-observer analysis was for observers 1-3, and for the inter-observer analysis was for the three paired comparisons (Observer 1 to 2, 1 to 3 and 2 to 3).

This allowed us to easily estimate the posterior distribution of each grouping level (from the 3 estimated alphas), which correspond to the different grouping levels we were interested in.

#model

$agree_i \sim$ Bernoulli* $(p_i)$

$logit(p_i) = alpha_j$

$alpha_j \sim$ Normal (mu.alpha, sd.alpha) #3-level ‘j’ for the 3 observers

#priors

mu.alpha ~ Normal (0, 1000) #prior for the mean of ‘j’ levels

sd.alpha ~ Uniform (0,10) #prior for the standard deviation of ‘j’levels

* note that Bernoulli distribution is simply a Binomial with a sample of 1 at each observation level

**Appendix 1b:** Binomial model formulation including explanatory variables

As with Appendix 1a we used a 1/0 response variable for the analysis of intra-observer and inter-observer comparisons. Here we included explanatory variables to help explain the variation in the agreement scores as an addition to the estimates. In the example below we include beta estimates for the effect of the cat's age (age), the quality of the radiograph (quality) and the time taken to assess the radiograph during the first assessment (time). The subscript 'i' relates to the individual observations, and the subscript 'j' refers to the group-level effects of observer (for intra-observer analyses) or observer-pairs (for the inter-observer analyses).

#model

$agree_i$ ~ Bernoulli* ($p_i$)

logit ($p_i$) = $alpha_j$ + beta.age*$age_i$ + beta.quality*$quality_i$ + beta.time*$time_i$

$alpha_j$ ~ Normal (mu.alpha, sd.alpha) #3-level 'j' for the 3 observers

#priors

mu.alpha ~ Normal (0, 1000) #prior for the mean of 'j' levels

sd.alpha ~ Uniform (0,10) #prior for the standard deviation of 'j'levels

* note that Bernoulli distribution is simply a Binomial with a sample of 1 at each observation level

**Appendix 1c:** Multinomial model formulation for calculating FHD score probabilities conditional on the initial hip score given

Here the data were initially subsetted into the assessment scores provided by other observers, given that a focal observer recorded a specific initial score. For example, when examining the conditional probabilities of the different subsequent FHD scores being given in a repeated (or second opinion) assessment, given that the initial assessment of any hip was '1', we found all hips that were scored by at least one person as having scored it a '1' and restricted our analysis to these hips for the FHD=1 analysis. All the other scores that were given by observers were then used in the multinomial analysis as a vector of possible subsequent FHD scores that could be observed at a 'future' time (either as a reassessment by the same person or by a different person in a second opinion). Thus, for a hip that had received a score of '1' by an observer and then all subsequent observations of that hip were one '0' and two '1's, the row of data would look like: 1, 2, 0, 0 (for 1 zero, 2 ones, 0 twos and 0 threes). Note that **bold type** indicates a vector of data or estimates instead of a single data point / parameter estimate.

The modelling then used took this vector of possible scores for each individual hip as the response variable and estimated the probability of each possible FHD score being given based on the total number of scores given (here 'total scores' = 3).

#model

$$\mathbf{Subsequent\ FHD\ score}_i \sim \text{Multinomial}\,(\mathbf{p}, \text{total scores}_i)$$

#multivariate prior

$$\mathbf{p} \sim \text{Dirichlet}\,(0.1, 0.1, 0.1, 0.1)$$
