# Supplementary material for: Repeatability of radiographic assessments for feline hip dysplasia suggest consensus scores in radiology are more uncertain than commonly assumed
Source: Sci Rep. 2022 Aug 17;12:13916. doi: 10.1038/s41598-022-18364-9 (PMC9385612; doi:10.1038/s41598-022-18364-9)
Supplement: Supplementary file 3 — Supplementary Information 3. [file 41598_2022_18364_MOESM3_ESM.pdf]

Supplementary Tables for the manuscript:

Repeatability of radiographic assessments for feline hip dysplasia suggest consensus scores in radiology are more uncertain than commonly assumed

By Elisabeth Ball, Margareta Uhlhorn, Per Eksell, Ulrika Olsson, Åsa Ohlsson & Matthew Low

In this file are the following Tables

Table S1a: Intra-observer GLMM model coefficient estimates

Table S1b: Inter-observer GLMM model coefficient estimates

Table S1c: Individual to mutual consensus GLMM model coefficient estimates

Table S2: Probabilities of changes in hip assessment scores based on a ‘median value’ consensus

Table S1a: Model posterior distribution coefficient estimates for the logit-link binomial GLMM estimating the impact of explanatory variables on intra-observer repeatability (see Fig. 2a).

The alpha estimates relate to the random effect levels representing each individual observer (observers 1-3 for alphas 1-3) and the beta estimates from the model relate to the: (1) age = effect of cat age (in days), (2) quality = the 'quality' of the radiograph (as a 1/0 variable related to whether the radiograph had the right contrast and the cat positioned correctly in the radiograph), and (3) time = the time (in seconds) that an observer spent assessing the radiograph before making a decision during the first assessment. See Appendix S1 for a formal description of the model formulations.

The estimates below are the (logit-link) means and SDs of the posterior distributions from the Bayesian model formulation. The Pr(effect) is the probability calculated from the posterior distribution that the direction of the effect goes contrary to the estimated direction, and can be roughly considered a type of p-value for estimating the confidence in the effect (i.e. we can be 99.92% confident that the time taken increases the intra-observer variability in assessments).

	Mean	SD	Pr(effect)
alpha[1]	0.6229	0.1590	
alpha[2]	0.7783	0.1671	
alpha[3]	1.030	0.1782	
age	-0.000098	0.0004	0.395
quality	0.034	0.023	0.538
**time**	**-0.0166**	**0.0053**	**0.0008**

Table S1b: Model posterior distribution coefficient estimates for the logit-link binomial GLMM estimating the impact of explanatory variables on inter-observer repeatability (summarised from assessments 1 & 2).

The alpha estimates relate to the random effect levels representing each individual observer (observers 1-3 for alphas 1-3) and the beta estimates from the model relate to the: (1) age = effect of cat age (in days), (2) quality = the 'quality' of the radiograph (as a 1/0 variable related to whether the radiograph had the right contrast and the cat positioned correctly in the radiograph), and (3) time = the time (in seconds) that an observer spent assessing the radiograph before making a decision during the first assessment. See Appendix S1 for a formal description of the model formulations.

The estimates below are the (logit-link) means and SDs of the posterior distributions summarised from the Bayesian model formulation from both assessments. The Pr(effect) is the probability calculated from the posterior distribution that the direction of the effect goes contrary to the estimated direction, and can be roughly considered a type of p-value for estimating the confidence in the effect (i.e. we can be 99.998% confident that the time taken increases the between-observer variability in assessments).

	Mean	SD	Pr(effect)
alpha[1]	0.901	0.101	
alpha[2]	0.873	0.107	
alpha[3]	0.938	0.106	
age	-0.000031	0.0003	0.545
quality	0.026	0.16	0.439
**time**	**-0.0136**	**0.0037**	**0.00001**

Table S1c: Model posterior distribution coefficient estimates for the logit-link binomial GLMM estimating the impact of explanatory variables on individual assessment to mutual consensus repeatability (see Fig 2b).

The alpha estimates relate to the random effect levels representing each individual observer (observers 1-3 for alphas 1-3) and the beta estimates from the model relate to the: (1) quality = the 'quality' of the radiograph (as a 1/0 variable related to whether the radiograph had the right contrast and the cat positioned correctly in the radiograph), (2) time = the time (in seconds) that an observer spent assessing the radiograph before making a decision during the first assessment, (3) reading = a 0/1 variable describing whether the initial score was given during assessment 1 (reference category 0) or assessment 2, and (4) the interaction between reading and time. See Appendix S1 for a formal description of the model formulations.

The estimates below are the (logit-link) means and SDs of the posterior distributions summarised from the Bayesian model formulation from both assessments. The Pr(effect) is the probability calculated from the posterior distribution that the direction of the effect goes contrary to the estimated direction, and can be roughly considered a type of p-value for estimating the confidence in the effect (i.e. we can be 92.7% confident that the reading*time interaction shows the effect of time being greater in the first assessment compared to the second).

	Mean	SD	Pr(effect)
alpha[1]	0.640	0.135	
alpha[2]	0.436	0.146	
alpha[3]	1.124	0.158	
quality	-0.218	0.176	0.134
**time**	**-0.019**	**0.005**	**0.000**
**reading**	**1.203**	**0.153**	**0.000**
**reading*time**	**0.011**	**0.007**	**0.073**

Table S2: Probabilities of getting an FHD score in a second assessment, conditional on the FHD score given in the first assessment (Original = 0, 1, 2 or 3) for a 'median' consensus score (i.e. at least 2 out of 3 radiologists agree on the score, or if they all disagree, then using the middle score as the 'consensus'). Probabilities for all possible outcomes are presented as the mean and 95% Bayesian CIs as generated from multinomial modelling of the data collected in this study. The left column shows the FHD score in the first and second assessments: e.g. if a hip was scored 0 in the first assessment, and 2 in the second assessment, this is represented as 0 → 2. The probability of agreement between the first and second assessment is highlighted in grey for each possibility.

FHD score	Median Consensus	
	Mean	95% CI
Original = **0**		
0 → 0	0.836	0.71 - 0.93
0 → 1	0.138	0.05 - 0.26
0 → 2	0.023	0 - 0.08
0 → 3	0.001	0 - 0.01
Original = **1**		
1 → 0	0.052	0.01 - 0.11
1 → 1	0.679	0.57 - 0.77
1 → 2	0.267	0.17 - 0.37
1 → 3	0.001	0 - 0.006
Original = **2**		
2 → 0	0.024	0.0 - 0.08
2 → 1	0.166	0.07 - 0.29
2 → 2	0.690	0.54 - 0.82
2 → 3	0.118	0.04 - 0.23
Original = **3**		
3 → 0	0.002	0 - 0.03
3 → 1	0.003	0 - 0.04
3 → 2	0.005	0 - 0.05
3 → 3	0.987	0.91 - 1
